# Cool but Counterproductive: Interactive, Web-Based Risk Communications Can Backfire

**DOI:** 10.2196/jmir.1665

**Published:** 2011-08-25

**Authors:** Brian J Zikmund-Fisher, Mark Dickson, Holly O Witteman

**Affiliations:** ^1^Department of Health Behavior and Health EducationSchool of Public HealthUniversity of MichiganAnn Arbor, MIUnited States; ^2^Division of General MedicineDepartment of Internal MedicineUniversity of MichiganAnn Arbor, MIUnited States; ^3^Center for Bioethics and Social Sciences in MedicineMedical SchoolUniversity of MichiganAnn Arbor, MIUnited States; ^4^Risk Science CenterSchool of Public HealthUniversity of MichiganAnn Arbor, MIUnited States

**Keywords:** Patient-provider communication

## Abstract

**Background:**

Paper-based patient decision aids generally present risk information using numbers and/or static images. However, limited psychological research has suggested that when people interactively graph risk information, they process the statistics more actively, making the information more available for decision making. Such interactive tools could potentially be incorporated in a new generation of Web-based decision aids.

**Objective:**

The objective of our study was to investigate whether interactive graphics detailing the risk of side effects of two treatments improve knowledge and decision making over standard risk graphics.

**Methods:**

A total of 3371 members of a demographically diverse Internet panel viewed a hypothetical scenario about two hypothetical treatments for thyroid cancer. Each treatment had a chance of causing 1 of 2 side effects, but we randomly varied whether one treatment was better on both dimensions (strong dominance condition), slightly better on only one dimension (mild dominance condition), or better on one dimension but worse on the other (trade-off condition) than the other treatment. We also varied whether respondents passively viewed the risk information in static pictograph (icon array) images or actively manipulated the information by using interactive Flash-based animations of “fill-in-the-blank” pictographs. Our primary hypothesis was that active manipulation would increase respondents’ ability to recognize dominance (when available) and choose the better treatment.

**Results:**

The interactive risk graphic conditions had significantly worse survey completion rates (1110/1695, 65.5% vs 1316/1659, 79.3%, *P* < .001) than the static image conditions. In addition, respondents using interactive graphs were less likely to recognize and select the dominant treatment option (234/380, 61.6% vs 343/465, 73.8%, *P* < .001 in the strong dominance condition).

**Conclusions:**

Interactivity, however visually appealing, can both add to respondent burden and distract people from understanding relevant statistical information. Decision-aid developers need to be aware that interactive risk presentations may create worse outcomes than presentations of static risk graphic formats.

## Introduction

Ample evidence exists that even highly educated adults can have poor numeracy skills [1-4]. As a result, patient decision aids and other patient communications that incorporate risk statistics will only be effective in improving patient decision making if they use design features that make these risk communications easier to understand. In this vein, researchers have evaluated the benefits of giving people more intuitive representations of risks by, for example, using frequencies instead of percentages [5-8] and testing a wide variety of visual displays, such as bar graphs, pie charts, and pictographs or icon arrays [9-14].

Improving risk-communication methods is also important because patients are likely to translate risk statistics into intuitive “gist” representations [15,16] that may influence anxiety or worry, powerful emotions that have significant impacts on people’s responses to health risks and disease [17-22]. In fact, emotional responses may mediate cognitive risk perceptions or shape behavior independently, or both [23-25].

Recently, Natter and Berry suggested that communications that force the audience to actively process risk information may be more effective than more passive displays [26]. In their study, participants were better calibrated in their perceptions of medication side effects when they created a bar graph of the risk instead of just viewing one. Similarly, Ancker and colleagues found that a Web-based, game-like, interactive risk graphic in which participants clicked in a matrix until they uncovered a risk event had the effect of reducing disparities in risk perceptions between high- and low-numeracy participants [27]. While such interactive graphing tasks were formerly difficult to implement, advances in the interactive capabilities of the Internet provide an opportunity to integrate interactive graphing tasks into patient decision-support materials. Such exercises could be seen as one method of increasing patients’ active processing of risk information. Indeed, Ancker and colleagues also showed that their interactive risk graphics may elicit emotional responses that reflect the potential for increased understanding of actual risks and better ability to compare and contrast risks [28].

We therefore hypothesized that a task designed for active processing might help patients not only to comprehend the statistics presented to them but also to integrate these facts into their decision making and thereby make better decisions.

To test the hypothesis that interactive graphing tasks could improve knowledge of risk information and decision making relevant to the risks, we conducted an Internet-administered experiment in which participants read a hypothetical treatment decision-making scenario that included information about the risks of 2 possible side effects. While some study participants passively viewed the risk information in state-of-the-art static risk images, others had to actively complete an interactive graphing task that asked them to fill in an icon array to match risk statistics that were provided numerically. We assessed whether the interactive task affected survey completion rates, treatment choices, and gist knowledge about the treatment options.

## Methods

### Recruitment

A stratified random sample of US adults age 21 years and older was selected from a panel of Internet users administered by Survey Sampling International (Shelton, CT, USA). To ensure at least moderate demographic diversity (but not representativeness) and offset large expected variations in response rates, we drew distinct subsamples by both age and race, and dynamically adjusted the number of email invitations in each demographic subsample until all quotas were achieved. Selected panel members received email invitations to complete the online survey. Upon completing the survey, participants were entered into both an instant-win contest and a monthly drawing administered by Survey Sampling International for modest prizes.

### Design of the Study

Respondents read a short vignette in which they imagined being diagnosed with thyroid cancer and discussing treatment options with their doctor. The vignette discussed 2 types of radiation treatment, external beam therapy and seed therapy, which were described as being equally effective in treating the patient’s type of thyroid cancer. Both therapies were described as having a chance of causing 2 side effects: (1) fatigue, and (2) mouth and throat problems. We chose thyroid cancer as the disease context because its comparative unfamiliarity (versus, for example, breast or prostate cancer) meant that few study participants would be likely to have preconceived beliefs about treatment options or their associated risks.

Our primary research question was to determine whether an interactive graphing task would increase respondents’ ability to recognize a better treatment option (ie, one with lower risks of side effects) when such was available. To do so, we experimentally varied the content of the 2 survey pages that presented the risk of each side effect with the two treatment options. On those pages, we varied 2 factors in a 3 (risk levels) × 2 (graphic type) between-subjects design.

**Table 1:** Side-effect risks presented in the hypothetical vignette

	Risk of fatigue		Risk of mouth or throat problems	
	Beam therapy	Seed therapy	Beam therapy	Seed therapy
Trade-off condition	12%	11%	13%	15%
Mild dominance condition	12%	11%	15%	15%
Strong dominance condition	12%	11%	21%	15%

The risk-level manipulation is summarized in Table 1. By varying the likelihood of experiencing mouth or throat problems with beam therapy, we created 3 distinct decision contexts: (1) a trade-off condition, in which each treatment had a higher likelihood of one side effect and a lower likelihood of the other, (2) a mild dominance condition, in which seed therapy had a very slightly lower rate of fatigue and the same rate of mouth and throat problems, and (3) a strong dominance condition in which seed therapy had a lower rate of both side effects.

**Figure 1:**
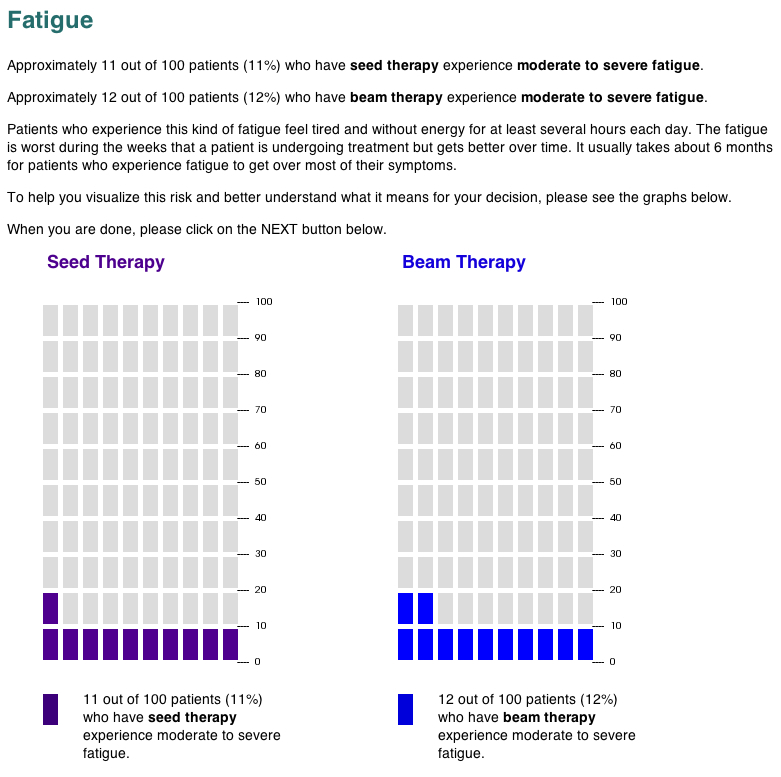
Screen image of the static pictographs

**Figure 2:**
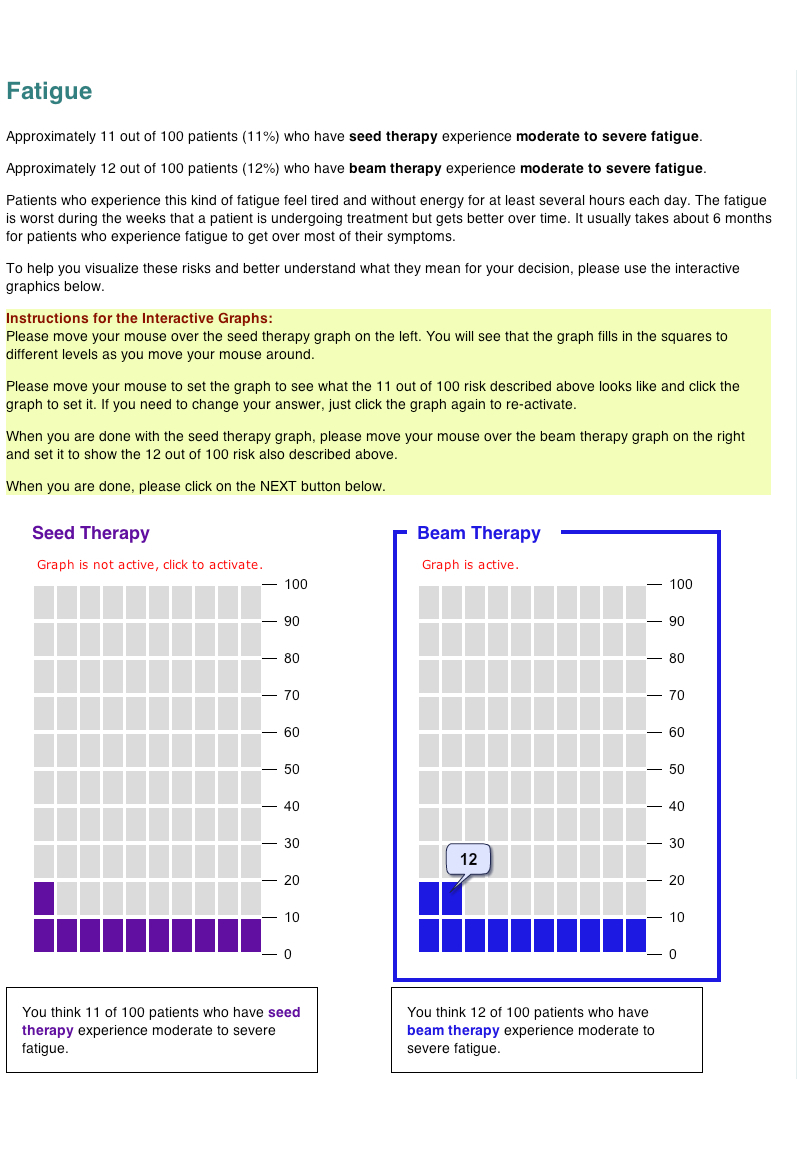
Screen image of the interactive graphing task

All risk information for the side effects was presented both numerically and in side-by-side graphics on sequential screens (first fatigue and then mouth and throat problems). Half of study participants viewed the risks displayed in static 100-unit icon arrays (also called pictographs: Figure 1), a format demonstrated to improve risk communication in a variety of medical contexts [9,13,29-33]. The remaining participants received an interactive version of the same icon array format (Figure 2), programmed in Flash (Adobe Systems Inc, San Jose, CA, USA). In the interactive exercise, the risk information was provided numerically but the graphics were initially blank (ie, all gray blocks). Participants were then instructed to use their mouse to click and/or drag in each graph to set it to the appropriate risk level. The graph continually adjusted to provide both visual and numerical feedback. (See Multimedia Appendix 1 for a movie demonstrating the interaction.) Participants were required to interact with and set the first of the 2 graphs on the page before they were allowed to go on in the study. The specific instructions given to participants are shown in Figure 1. Note, however, that participants were not prevented from advancing if they did not set the second graph or were inaccurate in their graphing of the risks; minimal use of the interactive graphics was sufficient to allow them to continue in the study. This design received Institutional Review Board exempt status approval as anonymous survey research.

### Measures and Covariates

Our primary outcome measure was the preferred treatment choice (beam or seed). We also asked respondents 2 gist knowledge questions in which they were to indicate which therapy had a higher risk of each of the side effects. In addition, we electronically tracked 2 measures of respondent burden: (1) time spent reading the static graphs pages or interacting with the interactive graphs pages, and (2) survey break-offs (to assess whether the interactive task annoyed people sufficiently to make them stop taking the survey).

Because individuals vary in their numeracy (ie, their facility and comfort with quantitative health information such as risk statistics), all study participants also completed the Subjective Numeracy Scale (SNS) [34]. The SNS is a validated measure of quantitative ability and of preferences for receiving information in numerical form that has previously been shown to correlate with the ability to recall and comprehend both textual and graphical risk communications [32,35,36]. A participant’s SNS score is calculated as his or her mean rating across the 8 SNS questions and ranges from 1 (least numerate) to 6 (most numerate). We also assessed participants’ level of education, which we model for analysis purposes as a 3-level variable: high school or less, some post-high school education but no bachelor’s degree, and bachelor’s degree or more, as well as standard demographics measures.

### Statistical Analyses

We used chi-square tests of proportions to test whether graph type affected treatment choices, knowledge recall, and survey discontinuation rates across the 3 risk-level conditions and logistic regression models that included both design factors, an interaction term, SNS score, and education level to assess the impact of these covariates on treatment choices. Because time spent on a given survey page has a highly skewed distribution, we then used Wilcoxon rank-sum tests to compare the distributions of time spent reading the static risk graphics versus completing the interactive graphing task. All analyses were performed using Stata (release 11; StataCorp LP, College Station, TX, USA), and all tests of significance were 2-sided and used alpha = .05.

## Results

### Recruitment

In total, 3371 people age 21 years and older reached the survey website and viewed the first content page. Of these, 17 reported having been actually diagnosed with thyroid cancer and were excluded as having pre-existing knowledge of the relevant treatment options, leaving 3354 possible participants.

Overall, 2426 (72%) of participants completed the entire survey, including questions on demographics that came toward the end of the survey instrument. Characteristics for those participants who answered each demographic question are reported in Table 2. We observed a wide range of educational achievement, with 889 participants (36.7%) having a bachelor’s or higher college degree but also 441 (18.2%) having completed only high school or less education. The SNS numeracy measure showed high reliability (Cronbach alpha = .84). Mean SNS score was 4.63 (SD 1.0), with substantial variation (range 1.75–6.0). Because questions about participant demographics came at the end of the survey, we cannot know whether the demographics of those who dropped out differ from those who completed the survey.

### Statistical Analyses

#### Dropout Rates and Time Spent on Risk Graphic Pages

As shown in Table 3, participants randomly assigned to complete the interactive graphics task were significantly less likely to complete the survey than those randomly assigned to view static graphs (65.5% vs 79.3%). More detailed examination confirmed that this difference was specifically due to the interactive graphics task. Over 23% of participants in the interactive graphics condition dropped out of the survey during that section of the survey, while less than 4% of participants viewing static graphics dropped out on those pages. Even among those who did progress beyond that point in the survey, it took significantly longer to complete the interactive graphing task than to view the static graphs.

**Table 2:** Participanta demographic characteristics

Characteristic		Distribution	Mean (SD)
**Age (years)**			49.1 (16.3)
	21–29	351 (14.5%)	
	30–39	448 (18.5%)	
	40–49	435 (18.0%)	
	50–59	396 (16.3%)	
	60–69	528 (21.8%)	
	≥70	266 (11.0%)	
**Gender**			
	Male	1212 (50.0%)	
	Female	1211 (50.0%)	
**Ethnicity**			
	Hispanic (any race)	269 (11.2%)	
**Race**^b^			
	White	2008 (82.7%)	
	African American	236 (9.7%)	
	All other	213 (8.8%)	
**Education**			
	≤ High school	44 (1.8%)	
	High school only	397 (16.4%)	
	Some college/trade	1093 (45.1%)	
	Bachelor’s degree	593 (24.5%)	
	Master’s/doctorate	296 (12.2%)	
**Subjective numeracy scale (mean rating)**			4.63 (1.00)
	1.00–1.99	45 (1.8%)	
	2.00–2.99	174 (6.8%)	
	3.00–3.99	500 (19.6%)	
	4.00–4.99	894 (35.1%)	
	5.00–5.99	862 (33.8%)	
	6.00	72 (2.8%)	

^a^ Reports results only for those respondents who completed each question or measure.

^b^ Respondents could mark more than 1 race.

**Table 3:** Survey completion times rates, by graphic type

	Static graphs	Interactive graphs	Significance
Discontinued survey at the risk graphic pages	58/1659 (3%)	391/1695 (23.1%)	χ^2^_1_ = 277; *P* < .001
Median time spent on the 2 pages with risk graphics (seconds)^a^	52	155	*z* = 33.63; *P* < .001
Completed entire survey	1316/1659 (79.3%)	1110/1695 (65.5%)	χ^2^_1_ = 80.2; *P* < .001

^a^ Among respondents who did not discontinue at the risk graphic pages.

#### Treatment Choices

**Figure 3:**
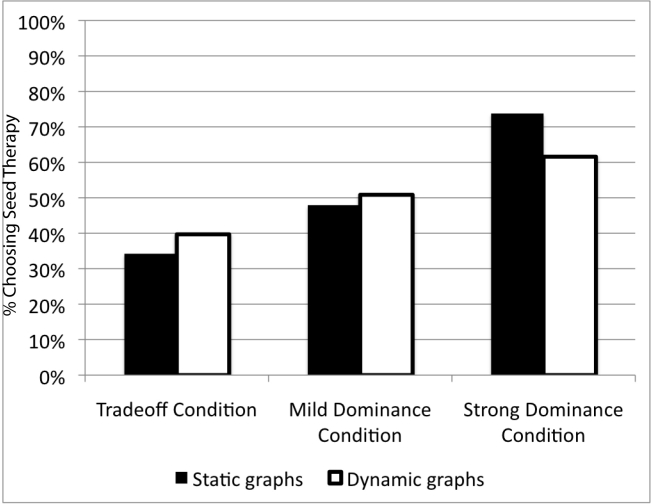
Treatment choices, by risk level and graph type conditions. Note: graph reports choices among those participants who completed the survey. Selection of seed therapy is a dominant choice in the mild and strong dominance conditions but not in the trade-off condition

However problematic, the longer completion times and lower completion rates noted above for participants completing the interactive graphing task might be acceptable if it resulted in improved treatment choices among those who did complete the task. Unfortunately, such was not the case. As shown in Figure 3, selection of the best (dominant) treatment option of seed therapy in the strong dominance condition was significantly higher among those in the static graphics group than in the interactive graphics group (343/465, 73.8% vs 234/380, 61.6%, χ^2^_1_ = 14.3, *P* < .001, among only those participants who answered the choice question; 343/536, 64.0% vs 234/558, 41.9%, χ^2^_1_ = 53.4, *P* < .001, in an intent-to-treat analysis that included all participants, including those who dropped out of the survey). There was no significant difference in treatment choices in the mild dominance condition, and a mild trend toward less selection of seed therapy by participants viewing static graphs versus interactive graphs in the trade-off condition.

In fact, across all 3 risk-level conditions, the interactive graphics condition made those participants less sensitive to variations in the risk of mouth and throat problems than were participants in the static graphs condition. A logistic regression analysis that included both experimental factors, as well as participant numeracy and education, confirmed a significant graph type × condition interaction (χ^2^_2_ = 18.4, *P* < .001). In addition, participants with higher SNS scores were significantly more likely to choose the best treatment (odds ratio = 1.18 per step on the 1–6 scale, *z* = 3.78, *P* < .001), but participant education had no effect. More complicated models found no significant interaction between participants’ numeracy levels and the effect of graph type.

We also examined whether those participants in the interactive graphics condition who accurately graphed the risk statistics made different treatment choices from those who had difficulty completing this task. In the strong dominance condition (the only one that showed a significant difference overall between participants who viewed interactive and static graphics), study participants who accurately graphed all 4 side-effect risks (243, 64.0%, of the 380 who answered the treatment choice question) were significantly more likely to choose the dominant option of seed therapy (164/243, 67.5% vs 70/137, 51%, χ^2^_1_ = 10.0, *P* = .002). We note, however, that optimal decision making remained at somewhat lower levels than observed in the static graphics condition (164/243, 67.5% vs 343/465, 73.8%, χ^2^_1_ = 3.1, *P* = .08), thereby providing no evidence that even completely accurate use of an interactive graphing task would improve people’s treatment selections.

#### Gist Knowledge

As shown in Table 4, study participants who had to complete interactive graphing tasks were also less able to identify which treatment had the higher risk of mouth or throat problems in the trade-off condition (43.8% vs 51.6%). Logistic regression analysis found this effect only marginally significant (odds ratio = 0.77, *z* = –1.77, *P* = .08) when controlling for the highly significant effect of numeracy (odds ratio = 1.52 per step on the 1–6 scale, *z* = 5.17, *P* < .001). There were no significant differences in gist knowledge about mouth and throat problems in the other 2 conditions. Nor were there any significant differences in gist knowledge about which treatment had a higher rate of fatigue (a side effect that did not vary across the 3 risk-level conditions.)

**Table 4:** Percentage of participants correctly identifying which treatment had a higher rate of mouth and throat problems, by condition and graphic type

	Static graphs	Interactive graphs	Significance
Trade-off condition	225/436 (51.6%)	165/377 (43.8%)	χ^2^_1_ = 5.0; *P* = .03
Mild dominance condition	404/502 (80.5%)	338/414 (81.6%)	χ^2^_1_ = 0.2; *P* = .66
Strong dominance condition	288/452 (63.7%)	226/374 (60.4%)	χ^2^_1_ = 0.9; *P* = .33

## Discussion

### Principal Results

The promise of interactivity is to potentially encourage people not just to read relevant information but also to actively process it. Such active processing might help people to remember the information and use it to make better treatment decisions.

Unfortunately, the promise of interactive risk-communication exercises remained unfulfilled in our study. Our interactive task, which asked participants to graph pairs of risk statistics that were clearly presented in numerical format on the same page, increased survey dropouts and resulted in lower knowledge and poorer treatment choices than among those who viewed the information already displayed in equivalent static graphs.

### Limitations

Our study has several key limitations. First and foremost, we tested a single type of interactive task with a particular user interface, one that did not undergo external usability testing. It is certainly possible that other tasks or more intuitive interfaces could have improved the use and user experience of the interactive task and potentially thereby improved outcomes. Second, as noted above, we asked otherwise healthy adults to answer a hypothetical scenario in an Internet survey. As a result, it is likely that these participants were considerably less motivated to work out how to do the interactive task and to think about the risk information we provided than real patients would be when reading a detailed decision aid designed to inform them and help them make their own medical decisions. Yet, we observed no advantage of the interactive task even for those participants who completed it perfectly. It is hard to imagine that even high levels of motivation would be enough to not just offset the knowledge and decision-making deficits we observed but actually accrue significant benefits from completing the interactive graphing exercise.

### Comparison with Prior Work

As Natter and Berry note, although other research exists that uses interactive graphics in risk communication [37], such research has not generally evaluated the interactive graphics against their passive counterparts. Accordingly, their study was the first to demonstrate the potential of interactive graphics over standard graphics by showing that people who draw a bar graph rather than simply viewed one had better recall of medication side effects [26]. More recent research has suggested that interactive risk graphics in which participants explore a risk matrix until they find a risk event could also reduce numeracy disparities in risk perceptions [27].

By contrast, our study showed no such positive effect. In fact, participants who were asked to interactively graph the risk information in our study did significantly worse on multiple outcome measures. This reversal may be partly accounted for by the fact that we used pictographs (icon arrays), which are less familiar to people than bar graphs, and also partly by the fact that our study was conducted online, whereas Natter and Berry’s used paper booklets. We also speculate post hoc that the novelty of the interactive task may have led participants to devote a majority of their attention and cognitive resources to figuring out what they were supposed to do. This effect may therefore have prevented our participants from engaging in the type of deeper, meaning-finding processing that we were hoping to stimulate. As noted above, a more in-depth, user-centered design process might have resulted in a better interface and hence reduced this problem.

Our different result may also derive from the fact that our task implemented a form of “teach-back” instructional methodology that encouraged participants to restate information they already had, whereas Ancker and colleagues’ research used an exploratory task that encouraged discovery of the risk itself. In qualitative analyses of focus group transcripts, Ancker and colleagues concluded that their interactive risk graphics elicited more emotional responses than static graphics did, with more participants expressing concern about large risks and/or relief about small ones [28]. Their subsequent experimental study found no overall effect of graphic type on risk estimates or risk feelings [27]. While our study did not ask participants about how the graphics made them feel about their risk, we did find that people were less likely to choose the clearly optimal option after having used an interactive risk graphic than they were with a standard risk graphic. This suggests that the increased emotional response observed by Ancker et al may be an artifact of their game-like task and that more straightforward types of interactivity may not lead to improved choices.

Research on other forms of interactivity in health education such as video games [38] and immersive 3-dimensional environments [39] has suggested caution in considering how these applications might or might not improve outcomes in health education. Our results complement these prior findings by showing that interactive approaches to risk communication should come with similar caveats.

### Conclusions

Interactive risk graphics are intriguing, but their use may be counterproductive to the purpose of effectively communicating to patients about health risks. While the movement of patient decision aids from booklet and DVD formats to online resources has created opportunities for greater technological sophistication, our research should serve as a cautionary tale to developers and educators seeking to ensure that their materials are “cutting edge.” Our teach-back interactive graphing task was intended to reinforce deeper cognitive processing of decision-relevant risk information, exactly the type of thinking most decision aids seek to promote in patients. Yet, in this case, our intervention backfired and created worse decision outcomes. More research is clearly needed to evaluate different types of interactive risk communications and to identify the design features of interactive exercises that lead to better results versus the features of those that do not. In the meantime, decision-aid designers should proceed with caution when considering the use of flashy risk graphics.

## Multimedia Appendix 1

MP4 movie of the interactive graphing task.

## Abbreviations

SNS: Subjective Numeracy Scale
